# Mental Health Care Disparities Among US Pregnant Individuals in 2020–2021: A Cross-Sectional Study

**DOI:** 10.1007/s40615-024-02250-3

**Published:** 2024-12-17

**Authors:** Julisa Tindall, Monique J. Brown, Peiyin Hung

**Affiliations:** 1 South Carolina SmartState Center for Healthcare Quality, Arnold School of Public Health, University of South Carolina, Columbia, SC USA; 2 College of Social Work, University of South Carolina, Columbia, USA; 3 Rural and Minority Health Research Center, Arnold School of Public Health, University of South Carolina, Columbia, USA; 4 Department of Epidemiology and Biostatistics, Arnold School of Public Health, University of South Carolina , Columbia , USA; 5 Department of Health Services Policy and Management, Arnold School of Public Health, University of South Carolina , Columbia , USA; 6 USC Big Data Health Science Center, University of South Carolina, Columbia, USA

**Keywords:** Mental health, Healthcare disparities, Pregnant individuals, Rurality, Maternal race/ethnicity

## Abstract

**Purpose:**

To examine maternal characteristics associated with perceived unmet mental health needs and mental health care settings, focusing on residential rurality and race/ethnicity.

**Methods:**

This cross-sectional study analyzed self-reported unmet mental health needs and mental health care settings among 1097 pregnant respondents in the 2020–2021 National Survey on Drug Use and Health, incorporating the complex sampling weights for national representativeness.

**Findings:**

Non-Hispanic Black pregnant individuals and those living in nonmetro rural areas reported lower odds of unmet mental health needs compared to those in large metro areas and non-Hispanic White individuals. Pregnant individuals in nonmetro rural areas and non-Hispanic other pregnant individuals also reported lower odds of utilizing virtual mental care services, while non-Hispanic other pregnant individuals were less likely to receive prescription medication than their non-Hispanic White counterparts.

**Conclusions:**

Disparities in mental health care access by rurality and race/ethnicity reveal increased barriers for nonmetro rural and minority pregnant populations, particularly regarding virtual and prescription-based care. The lower unmet health needs among Black pregnant individuals and those living in nonmetro rural areas may reflect adjusted expectations or reliance on informal support systems, emphasizing the need to understand these perceptions. COVID-19’s impact on access patterns further highlights the need for more research on barriers to maternal mental health treatment. Tailored mental health interventions and policy reforms are needed to enhance accessible, culturally sensitive maternal mental health services across diverse communities.

## Introduction

Mental health during pregnancy is critical but often overlooked in maternal health care. Pregnant individuals’ emotional and psychological well-being can significantly impact their health and infant health outcomes. Maternal mental health issues, such as anxiety, perinatal and postpartum depression, and birth-related PTSD, are prevalent during pregnancy and childbirth, impacting one out of every five birthing individuals [1]. In the United States, from 2017 to 2019, mental health issues were the leading cause (23%) of pregnancy-related deaths [2]. Despite its prevalence, research has documented disparities in mental health care access, utilization, and outcomes among pregnant individuals [3–5]. However, data on the patterns of mental health care settings across pregnant women across geography, race, and ethnicity groups remain limited.

Access to timely mental health care is essential to prevent detrimental outcomes, yet access to care, often measured by health care infrastructure, resources, and provider availability, varied significantly by residential location/rurality (e.g., urban/rural), creating possible barriers for rural pregnant individuals in need [6]. It is estimated that around 65% of rural United States counties do not have psychiatrists, and more than 60% of individuals in these rural counties live in areas officially designated as experiencing shortages of mental health care providers [7]. The odds of the perinatal depression risk were 21% higher among rural women versus urban women, suggesting that rural pregnant women may face unique health care barriers that could contribute to this increased risk [8]. In addition to residential rurality, structural racism often leads to disproportionate barriers to health care access across racial and ethnic groups [9].

Indeed, studies have shown disparities in maternal mental health outcomes based on race and ethnicity [10, 11]. In the United States, major depressive disorder and other mental health issues during pregnancy and postpartum have a disproportionate impact on Black, Indigenous, and people of color (BIPOC) individuals [12]. One in eight birthing individuals is affected by postpartum depression (PPD), and the risk of PPD is 1.6 times higher for non-Hispanic Black than non-Hispanic White birthing individuals [13]. Despite this higher risk, BIPOC individuals have lower odds of receiving perinatal mental health treatment compared to White individuals [11]. These data highlight the need to explore the unique experiences and needs of diverse pregnant populations related to their mental health.

BIPOC pregnant individuals may face compounded challenges due to both geographic isolation and systemic barriers related to race. While an emerging body of research sheds light on the racial disparities in maternal mental health care, little is known about how these disparities vary by residence location [4, 10, 11]. The intersectionality of race and rurality is likely to heighten disparities in maternal mental health care. Research by Ceballos et al. highlighted racial/ethnic disparities in the prevalence of postpartum depression, with findings showing that compared to their White counterparts in large cities, Latino and Black individuals experience significantly higher rates of PPD. Specifically, in large urban areas, the disparities are pronounced, but these risks become even more acute when considering the living situations of Latino and Black individuals in small cities and rural communities. In these less urbanized rural areas, the risk of postpartum depression was found to be 40% greater in Latino individuals and 80% greater for Black individuals when compared to White individuals, underscoring the compound vulnerabilities associated with both race/ethnicity and geographic location [14].

The COVID-19 pandemic further intensified the demand for maternal mental health services while exacerbating access disparities. Pregnant individuals faced heightened mental health challenges due to pandemic-related stressors such as isolation, economic insecurity, and health concerns [15, 16]. In addition, health care resources were often diverted to address COVID-19 needs, which temporarily limited access to non-emergency mental health services. With the shift to telehealth during the pandemic, virtual care became an increasingly common option for addressing maternal mental health needs [17, 18]. However, rural and underserved populations often face additional challenges in accessing virtual care, including limited broadband availability and digital literacy barriers [19, 20].

While previous research has documented racial disparities in mental health care access among BIPOC populations [10, 11], a gap exists in understanding how these disparities intersect with rurality, especially regarding perceived unmet mental health needs and specific care settings like virtual care and prescription medication use. This study provides novel insights by examining these unique intersections, identifying disparities not only in access to care but also in the perceived adequacy and suitability of mental health services. By focusing on these specific dimensions, our findings can inform interventions that address both geographic and racial/ethnic barriers, particularly for underserved populations, and contribute to intervention research and policy development to promote equitable mental health care access for all pregnant individuals and improve maternal and infant health outcomes.

Using data from the 2020–2021 National Survey on Drug Use and Health (NSDUH), this study examined self-reported unmet mental health needs and mental health care settings among pregnant individuals by residential rurality types and race/ethnicity. Specifically, we hypothesize that there will be associations between maternal characteristics and unmet mental health needs (hypothesis 1) and that distributions in mental health care settings will vary by maternal residence rurality and race/ethnicity (hypothesis 2). Additionally, we hypothesize that the intersection of race/ethnicity and rurality will further influence both unmet mental health needs and mental health care settings (hypothesis 3), with disparities expected to be more pronounced in rural BIPOC populations. These hypotheses provide a broad framework for examining how various settings and maternal characteristics influence mental health care experiences in this population.

## Methods

### Data Source

A secondary analysis of publicly available, de-identified data from the 2020–2021 NSDUH was conducted. The original 2021 NSDUH dataset was used before its revision in January 2024. The study was reviewed and approved by the Institutional Review Board of the University of South Carolina reviewed and approved this study (ID: Pro00132044). The NSDUH is a national cross-sectional survey funded by the Substance Abuse and Mental Health Services Administration (SAMHSA). This survey provides information about mental health and substance use problems along with treatment for mental health and substance use disorders among individuals 12 years old or older in the United States [21, 22]. However, this analysis focused exclusively on perceived unmet mental health needs and mental health treatment among pregnant individuals, excluding substance use treatment and individuals with substance use disorders from the analysis. The sample for this study was restricted to pregnant individuals aged 18 and older in the United States (*N* = 1097).

### Measures

Pregnant individuals who participated in the NSDUH were classified based on their self-report of pregnancy status (yes/no). The two main variables in this study are race/ethnicity and residential rurality. Race/ethnicity was categorized into four groups: Hispanic, non-Hispanic White, non-Hispanic Black/African American, and non-Hispanic other (i.e., Native American/Alaska Native, Native Hawaiian/Pacific Islander, Asian, and more than one race).

Residential rurality in this study was determined using the United States Department of Agriculture 2013 Rural/Urban Continuum Codes (RUCC), which are aligned with the classification system used by NSDUH [21–24]. The 2013 RUCC was selected to align with NSDUH’s original classification framework, ensuring consistency with the dataset’s 2020–2021 data collection period. This system categorizes counties into three types: large metropolitan, small metropolitan, and nonmetropolitan. Large metro counties represent areas with populations of 1 million or more, while small metro counties include areas with populations ranging from 250,000 to less than 1 million. Nonmetro counties encompass more rural areas, ranging from urban clusters of fewer than 20,000 to completely rural areas that are more isolated from metro centers. For broader interpretive purposes, “urban” represents a combination of large and small metro areas, while “rural” refers to nonmetro areas. This approach allows us to present detailed results while contextualizing them within a general urban–rural framework commonly used in health disparity research [23, 24].

The two primary outcomes examined were mental health needs and mental health care settings. Mental health need was measured as perceived unmet need for mental health care treatment, with or without receiving mental health treatment. Mental health care settings examined include receiving inpatient, outpatient, virtual, and prescription medication treatment (yes/no) in the past year [21, 22].

In examining the complex factors for disparities in maternal mental health care, this study drew upon the National Institute on Minority Health and Health Disparities (NIMHD) Research Framework [25]. This comprehensive framework was adapted to identify the multifaceted factors at different levels (i.e., individual, interpersonal, community, and societal) contributing to disparities in maternal mental health care access and utilization, focusing on minority health and health disparities. The identified covariates reflect the following sociodemographic: maternal age group (18–25, 26–34, and 35 or older), education (less than high school, high school graduate, some college/associates degree, and college graduate), health insurance (yes/no), employment status (employed/not employed), family income (less than $20,000; $20,000–$49,999; $50,000–$74,999; and $75,000 or more), and marital status (married/not married) [21, 22].

### Statistical Analysis

Statistical analysis was performed using the statistical software SAS Version 9.4 (SAS Institute Inc.). Logistic regression analyses were conducted to explore the association between the main variables (race/ethnicity and rurality) and outcomes (mental health needs and mental health care settings). Descriptive and bivariate statistics were also used to provide an overview of the data and initial relationships. Multivariable logistic regressions were conducted by simultaneously entering the aforementioned covariates with the main independent variables (race/ethnicity and rurality) and the outcome of mental health care settings to control for potential confounding effects. To examine the differential associations between maternal race/ethnicity and outcomes by rurality, additional multivariable models incorporated interactions of race/ethnicity and rurality. Considering the complex survey design and probability of sampling, a complex surveying weighing variable was included according to the guidelines of NSDUH to ensure the representativeness of the national population [21, 22]. *p*-values < 0.05 were considered statistically significant for all analyses. The imputed and recoded variables for sociodemographic, predictor, and outcomes variables were analyzed.

## Results

### Participants Characteristics

Participants’ characteristics by rurality can be found in Table 1 and by race/ethnicity in Table 2. The sample consisted of 1097 pregnant individuals living in the United States. Out of the 1097 pregnant individuals, 657 (52.7%) were non-Hispanic White (194 [18.9%] Hispanic, 127 [16.4%] non-Hispanic Black, 119 [12%] non-Hispanic other). The majority of participants resided in metro areas, with 444 (54.4%) in large metro areas and 428 (29.8%) in small metro areas, while only 225 (16.2%) lived in nonmetro areas (designated as rural in this analysis).

**Table 1:** Participants’ characteristics by rurality

Maternal characteristics	**All 2020–2021**		**Residential rurality**			*p*-values for differences in maternal characteristics by residential rurality
	Number of pregnant adults	Weighted Col%	Large metro	Small metro	Nonmetro	
			Number (weighted %) of pregnant adults			
Maternal race/ethnicity						
Non-Hispanic White	657	52.7	211 (42.7)	278 (62.3)	168 (69.0)	0.005
Non-Hispanic Black	127	16.4	65 (17.5)	43 (14.8)	19 (15.6)	
Hispanic	194	18.9	112 (24.8)	61 (14.3)	21 (7.4)	
Non-Hispanic other^a^	119	12	56 (15.0)	46 (8.6)	17 (8.0)	
Maternal age group						
18–25 years old	411	26.4	123 (17.6)	171 (35.6)	117 (39.1)	< 0.0001
26–34 years old	527	52.7	231 (53.5)	202 (50.8)	94 (53.8)	
35 or older	159	20.9	90 (28.9)	55 (13.6)	14 (7.1)	
Education						
Less than high school	123	12.9	44 (14.9)	44 (8.3)	35 (14.5)	0.004
High school grad	253	22	95 (17.7)	92 (23.9)	66 (33.0)	
Some college/associates degree	310	29.4	109 (26.1)	128 (31.8)	73 (36.0)	
College graduate	411	35.7	196 (41.3)	164 (36.0)	51 (16.5)	
Health insurance						
Yes	1017	91.8	408 (92.7)	399 (92.2)	210 (87.8)	0.59
No	80	8.2	36 (7.3)	29 (7.8)	15 (12.2)	
Employment status						
Employed	559	51.9	244 (48.3)	216 (53.0)	103 (37.8)	0.14
Not employed	538	48.1	200 (51.7)	212 (47.0)	122 (62.2)	
Family income						
Less than $20,000	223	21.2	78 (16.2)	85 (19.4)	60 (41.5)	0.001
$20,000–$49,999	309	28.9	117 (28.3)	128 (31.6)	64 (25.9)	
$50,000–$74,999	141	12.7	50 (11.8)	54 (14.9)	37 (12.1)	
$75,000 or more	424	37.2	199 (43.7)	161 (34.1)	64 (20.5)	
Marital status						
Married	676	58.7	267 (60.1)	268 (55.4)	141 (60.2)	0.59
Not married	421	41.3	177 (39.9)	160 (44.6)	84 (39.8)	

recoded variables were used. *p*-values were calculated using Rao-Scott chi-square test

^a^Non-Hispanic other includes Native American/Alaska Native, Native Hawaiian/Pacific Islander, Asian, and more than one race

**Table 2:** Participants’ characteristics by race/ethnicity

Maternal characteristics	All 2020–2021		Maternal race/ethnicity				*p*-values for differences in maternal characteristics by race/ethnicity
	Number of pregnant adults	Weighted Col%	Non-Hispanic White	Non-Hispanic Black	Hispanic	Non-Hispanic other^a^	
			Number (weighted %) of pregnant adults				
Residential rurality							
Large metro	444	54.4	211 (44.0)	65 (58.0)	112 (71.5)	56 (68.1)	0.005
Small metro	428	29.4	278 (34.8)	43 (26.6)	61 (22.2)	46 (21.2)	
Nonmetro	225	16.2	168 (21.2)	19 (15.4)	21 (6.3)	17 (10.7)	
Maternal age group							
18–25 years old	411	26.4	223 (24.7)	64 (35.1)	86 (32.3)	38 (12.8)	0.0003
26–34 years old	527	52.7	338 (59.6)	48 (50.4)	90 (43.5)	51 (40.4)	
35 or older	159	20.9	96 (15.7)	15 (14.5)	18 (24.2)	30 (46.8)	
Education							
Less than high school	123	12.9	47 (4.6)	26 (13.1)	40 (27.0)	10 (26.7)	< 0.0001
High school grad	253	22	128 (21.9)	43 (30.1)	62 (25.2)	20 (6.4)	
Some college/associates degree	310	29.4	165 (23.5)	43 (43.1)	68 (38.2)	34 (22.8)	
College graduate	411	35.7	317 (50.0)	15 (13.7)	24 (9.6)	55 (44.1)	
Health insurance							
Yes	1017	91.8	622 (94.8)	118 (91.0)	164 (80.2)	113 (97.7)	0.003
No	80	8.2	35 (5.2)	9 (9.0)	30 (19.8)	6 (2.3)	
Employment status							
Employed	559	51.9	388 (56.1)	40 (31.6)	67 (40.6)	64 (47.2)	0.02
Not employed	538	48.1	269 (43.9)	87 (68.4)	127 (59.4)	55 (52.8)	
Family income							
Less than $20,000	223	21.2	86 (12.2)	64 (45.4)	54 (31.6)	19 (11.3)	< 0.0001
$20,000–$49,999	309	28.9	157 (23.3)	38 (36.9)	81 (34.5)	33 (34.0)	
$50,000–$74,999	141	12.7	95 (15.6)	7 (3.7)	23 (10.7)	16 (15.7)	
$75,000 or more	424	37.2	319 (48.9)	18 (14.0)	36 (23.2)	51 (39.0)	
Marital status							
Married	676	58.7	495 (73.4)	19 (22.6)	88 (43.3)	74 (68.2)	< 0.0001
Not married	421	41.3	162 (26.6)	108 (77.4)	106 (56.7)	45 (31.8)	

recoded variables were used. *p*-values were calculated using Rao-Scott chi-square test

^a^Non-Hispanic Other includes Native American/Alaska Native, Native Hawaiian/Pacific Islander, Asian, and more than one race

In examining the nonmetro (rural) subset of the sample, non-Hispanic White participants were the largest group, with 168 individuals (69% of nonmetro residents). Other groups residing in nonmetro areas included 19 (15.6%) non-Hispanic Black individuals, 17 (8%) non-Hispanic other individuals, and 21 [7.4%] Hispanic individuals. About 119 (7.8%) of pregnant individuals reported having unmet mental health needs in the past year.

**Fig. 1:**
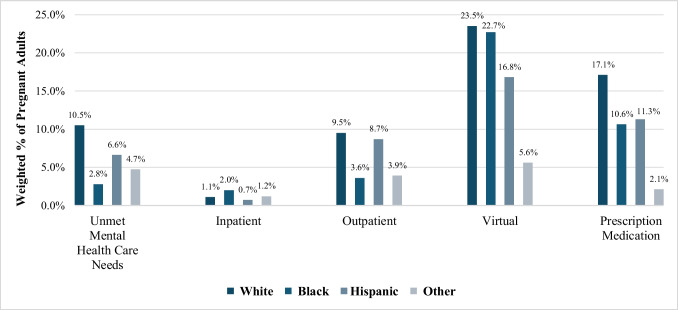
Mental health needs and health care services utilization by race/ethnicity. Notes: data represent weighted percentages of pregnant respondents from the 2020–2021 NSDUH. Percentages reflect reported unmet mental health needs and utilization of mental health services by race/ethnicity. Differences are based on observed percentages and are not tested for statistical significance

**Fig. 2:**
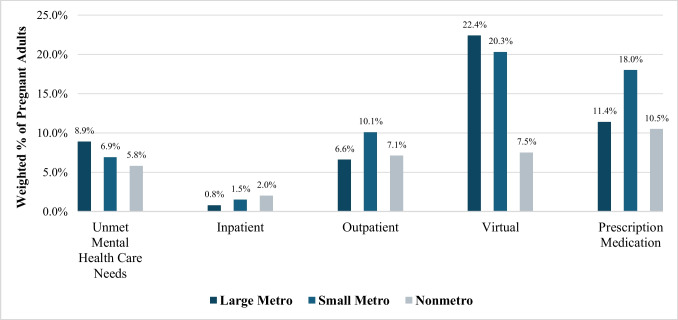
Mental health needs and health care services utilization by rurality. Notes: data represent weighted percentages of pregnant respondents from the 2020–2021 NSDUH. Percentages reflect reported unmet mental health needs and utilization of mental health services by residential rurality. Differences are based on observed percentages and are not tested for statistical significance

Figure 1 displays participants’ mental health needs and health care service utilization by race/ethnicity, showing notable differences in the reporting of unmet mental health needs and the utilization of different mental health services among racial and ethnic groups. Key observations include higher reported unmet mental health needs among non-Hispanic Black and other racial groups compared to non-Hispanic Whites and Hispanics. Figure 2 presents participants’ mental health needs and health care service utilization by rurality. The findings show that individuals in nonmetro areas reported lower rates of unmet mental health needs but also showed different patterns in the utilization of health care services, with notably less use of virtual health care options compared to those in large metro areas. All descriptive statistics for perceived unmet mental health needs and mental health care settings are in Appendix Table 1.

### Perceived Unmet Mental Health Needs

This logistic regression assessed factors associated with perceived unmet mental health needs among pregnant individuals (Table 3). After controlling for education, health insurance status, employment status, family income, and marital status, non-Hispanic Black pregnant individuals reported significantly lower odds of perceived unmet mental health needs than their non-Hispanic White counterparts (odds ratio [OR] = 0.18; *p* = 0.002; confidence interval [CI] = 0.06–0.53). Residential rurality showed statistically significant associations. Specifically, pregnant individuals living in nonmetro areas reported 54% lower odds of perceived unmet mental health needs than those living in large metro urban areas (OR = 0.46; *p* = 0.04; CI = 0.21–1.0).

**Table 3:** Logistic regression analysis of mental health care outcomes among pregnant individuals aged 18 years and older (*N* = 1097), NSDUH 2020–2021

	Perceived unmet mental health needs		Inpatient		Outpatient		Virtual		Prescription medication	
	Odds ratio 95% (CI)	*p*-values	Odds ratio 95% (CI)	*p*-values	Odds ratio 95% (CI)	*p*-values	Odds ratio 95% (CI)	*p*-values	Odds ratio 95% (CI)	*p*-values
Residential rurality										
Large metro	Reference		Reference		Reference		Reference		Reference	
Small metro	0.57 (0.29–1.11)	0.1	1.59 (0.25–9.97)	0.62	1.45 (0.71–2.95)	0.3	0.67 (0.31–1.48)	0.32	1.66 (0.86–3.20)	0.13
Nonmetro	0.46 (0.21–1.0)	0.04	1.46 (0.34–6.22)	0.6	0.99 (0.33–2.97)	0.99	0.29 (0.10–0.81)	0.02	1.12 (0.55–2.27)	0.75
Maternal race/ethnicity										
Non-Hispanic White	Reference		Reference		Reference		Reference		Reference	
Non-Hispanic Black	0.18 (0.06–0.53)	0.002	0.72 (0.15–3.58)	0.68	0.30 (0.07–1.40)	0.12	1.81 (0.55–5.96)	0.32	0.69 (0.24–2.0)	0.49
Hispanic	0.59 (0.22–1.60)	0.3	0.39 (0.09–1.65)	0.19	1.18 (0.37–3.83)	0.78	1.44 (0.44–4.70)	0.54	0.88 (0.32–2.41)	0.8
Non-Hispanic other^a^	0.44 (0.15–1.32)	0.14	0.96 (0.12–7.92)	0.97	0.42 (0.13–1.33)	0.14	0.18 (0.03–0.94)	0.04	0.10 (0.03–0.38)	0.001

recoded variables were used. *p*-values were calculated using Rao-Scott chi-square test

*NSDUH* National Survey on Drug Use and Health, *CI* confidence intervals

^a^Non-Hispanic other includes Native American/Alaska Native, Native Hawaiian/Pacific Islander, Asian, and more than one race

#### Mental Health Treatment Utilization

Results from the logistic regressions related to mental health treatment utilization are presented in Table 3. Residence rurality and maternal race/ethnicity did not show statistically significant associations with inpatient and outpatient mental health care utilization.

Pregnant individuals living in nonmetro areas reported 98% lower odds of using virtual mental health care than those living in large metro areas (OR = 0.02; *p* = 0.02; CI = 0.10–0.81). Non-Hispanic other pregnant individuals reported 82% lower odds of virtual mental health care use than their non-Hispanic White counterparts (OR = 0.18; *p* = 0.04; CI = 0.03–0.94).

Mental health prescription medication utilization did not significantly differ by residential rurality. However, non-Hispanic other pregnant individuals reported 90% lower odds of receiving prescription medication than their non-Hispanic White counterparts (OR = 0.10; *p* = 0.001; CI = 0.03–0.38). Pregnant individuals with health insurance also reported five times the odds of receiving this type of care than uninsured pregnant individuals (OR = 5.11; *p* = 0.02; CI = 1.31–19.99). Full logistic regression models can be found in Appendix Table 2.

Additional models testing interactions between rurality and race/ethnicity did not yield significant results, indicating that disparities in mental health care access persisted independently by rurality and race/ethnicity.

## Discussion

Using the 2020–2021 National Survey on Drug Use and Health (NSDUH) data, this cross-sectional study found some similarities and differences in pregnant individuals’ experiences of mental health care across the United States. By applying the National Institute on Minority Health and Health Disparities (NIMHD) Research Framework, we examined individual, interpersonal, community, and societal influences that shape maternal mental health care access and utilization among diverse groups [25].

Regarding unmet mental health needs, non-Hispanic Black pregnant individuals reported significantly lower odds of perceived unmet mental health needs than their non-Hispanic White counterparts. Additionally, pregnant individuals living in a nonmetro rural area reported significantly lower odds of perceived unmet mental health needs. On an individual level, this may suggest that perceived unmet mental health needs are shaped by individual factors such as personal expectations, health literacy, and awareness of mental health services [11, 25]. The relatively low proportion of perceived unmet mental health needs observed in our sample may indicate adequate access for some but could also reflect adjusted expectations or reliance on informal support systems among rural and racial/ethnic minority groups. The NSDUH’s reliance on self-reported data may further influence these perceptions. Future research is needed to further examine factors shaping these perceptions of unmet mental health needs and to assess whether they align with actual access and adequacy of mental health care services.

Despite disparities in unmet mental health needs among pregnant individuals, our study observed that the actual receipt of outpatient and inpatient care was similar across different racial/ethnic groups and residential settings, which diverges from some previous studies on maternal mental health disparities [26]. This similarity at the interpersonal level may reflect varying support systems, such as family and community networks, that influence how different groups perceive and utilize formal care [11, 25]. This discrepancy may reflect unique aspects of our sample from the 2020–2021 NSDUH, including the timing of data collection during the COVID-19 pandemic, which likely influenced health care utilization patterns. COVID-19 disrupted health care access nationwide, shifting to virtual formats and limiting in-person services, particularly in areas with fewer resources [17, 18]. On a community level, these shifts may have temporarily altered typical patterns of mental health care utilization, potentially affecting perceived unmet mental health needs and actual access to services, especially among marginalized and rural communities [25, 27].

In the societal context, the pandemic exacerbated existing health disparities, disproportionately impacting marginalized and rural communities due to structural barriers and heightened health risks [25, 28]. This context suggests that the lack of disparities in inpatient and outpatient use may be due to temporary adjustments or constraints during the pandemic rather than long-term trends. Further research should explore these associations to clarify potential differences and implications. This may also suggest that access to these traditional forms of care is somewhat consistent among the studied population.

The finding of similarity in access to care can be interpreted in several ways. First, mental health care is complex and highly individualized, requiring a range of services and approaches to meet the diverse needs of pregnant individuals from varying socio- and cultural backgrounds. Simply accessing care is not sufficient to fulfill mental health needs. For instance, quality of care, cultural or personal expectations, or awareness about mental health issues are all potential drivers for perceived mental health needs [4, 7]. At both the interpersonal and community levels, the observed similarity in access may also be influenced by factors such as stigma, limited health literacy, or previous negative health care experiences, which can lead certain groups to delay seeking care until it becomes critical [11, 25]. While this might superficially appear as parity in access, systemic barriers, such as limited culturally competent care and varying levels of provider availability, may still prevent some groups from accessing care that fully meets their needs [29]. Addressing these disparities goes beyond ensuring basic access; it necessitates a deeper examination of the factors contributing to unmet mental health needs and developing specific interventions that account for the multifaceted nature of mental health care in pregnant populations.

Additionally, it is essential to recognize that racial and ethnic groups face distinct systemic barriers in maternal mental health care. For example, non-Hispanic Black individuals often experience higher levels of medical mistrust due to historical injustice, which can deter them from seeking timely mental health care [30]. Also, Hispanic individuals may encounter language barriers and a lack of culturally competent services, which can hinder both access to and quality of mental health care they receive [31]. Furthermore, Native American and Alaska Native populations face unique geographic barriers as well as limited access to culturally sensitive mental health providers within the Indian Health Services [32]. Additionally, Asian individuals may experience cultural stigma around mental health that reduces their likelihood of seeking formal mental health services [33]. These disparities fall under societal-level influence, where historical and ongoing systemic discrimination creates barriers that impact trust in the health care system across diverse groups [25, 30–33]. These barriers highlight the importance of individualized maternal mental health care that respects and addresses the unique challenges faced by each racial and ethnic group. Community and societal approaches are critical here, including improving provider cultural competence, implementing language assistance programs, and establishing community-based support systems connecting patients with trusted figures in their communities [25]. These tailored approaches are essential for ensuring that maternal mental health services meet the specific needs of each group.

Moreover, significant disparities were identified in the utilization of virtual mental health care. Pregnant individuals living in nonmetro rural areas reported lower odds of using virtual mental health services compared to those in large metro areas. Similarly, non-Hispanic other pregnant individuals showed significantly lower odds of virtual care utilization compared to their non-Hispanic White counterparts. This disparity highlights community-level barriers linked to digital infrastructure limitations in rural settings and societal-level structural inequalities that perpetuate the digital divide [25, 34]. Cultural factors, including language barriers, cultural perceptions of mental health, and the availability of culturally sensitive services, may play a crucial role in the adoption of virtual mental health services [20, 25]. In terms of the digital divide, recent research indicates that the digital divide between rural and urban areas restricts the application and efficacy of mental health services due to rural areas not having the required technological resources like sufficient broadband network speed and digital literacy [19, 20].

These findings highlight the critical need for a thoughtful and equitable distribution of virtual mental health resources, especially to ensure that individuals in rural areas and those belonging to racial/ethnic minority groups have equal access to these services. Specifically, increased collaboration between health care providers, mental health experts, community organizations, and community-based outreach and education can help to improve this gap. At a societal level, policy reforms and expanded telehealth infrastructure are necessary to improve service accessibility, especially in underserved areas, addressing both community and societal structural barriers [19, 20, 25].

Regarding prescription medication for mental health issues, the study found no significant differences by residential rurality but noted racial/ethnic disparities. Non-Hispanic other pregnant individuals had notably lower odds of receiving mental health prescription medication compared to non-Hispanic White pregnant individuals, underlining the existence of barriers that affect racial and ethnic minority groups and the need for target interventions and policies [35]. Specifically, interventions should be implemented to improve health care provider training in cultural competence, enhance patient education regarding mental health treatment options, and implement policy reforms aimed at eliminating systemic barriers to equitable care.

Putting all these together, findings highlight the importance of understanding not just the availability of mental health services but also their perceived adequacy and suitability for different pregnant populations, especially in the context of rurality and race/ethnicity. The observation of lower unmet mental health needs in rural and non-Hispanic Black groups may initially appear counterintuitive, especially when considering the broader context of disparities in mental health services access and utilization [36]. However, for both non-Black individuals and individuals living in rural areas, these findings may reflect adjusted expectations due to limited services, reliance on informal support systems, and cultural factors such as stigma and mistrust of the health care system, which may lead to underreporting or alternative coping strategies [37, 38].

Enhancing the suitability of mental health care requires culturally competent, accessible, and community-aligned services that address the unique needs of these populations. Future research should further investigate how rurality and race/ethnicity shape maternal mental health disparities, focusing on barriers to treatment, care quality, and long-term outcomes. By addressing these aspects, mental health care can better meet the needs of all pregnant individuals.

### Limitations

This study highlights rurality and race/rurality as factors influencing mental health care accessibility and utilization among pregnant individuals; however, findings should be interpreted cautiously due to some limitations. First, the NSDUH measured pregnancy at the time of the interview but assessed perceived unmet mental health needs and treatment over the prior year, meaning it may not fully align with pregnancy periods. Additionally, this self-reported data may introduce recall bias and underreporting due to stigma surrounding mental health. While the NSDUH uses computer-assisted interviews to reduce bias, social desirability effects may still influence responses. Moreover, the overlap with the COVID-19 pandemic likely temporarily altered typical mental health patterns, especially for marginalized and rural communities, and these findings may not reflect long-term trends. Wide confidence intervals also suggest that there might be increased uncertainty and variability in the data, highlighting the need for more robust data collection methods. Lastly, smaller sample sizes prevented analysis of certain racial groups, such as Native American/Alaska Natives, Native Hawaiian/Pacific Islanders, Asians, and multiracial individuals, warranting future research focused on these populations.

## Conclusion

Through a secondary data analysis of the 2020–2021 survey data, this study sheds light on the complex variations in individual perceived unmet mental health needs and care settings across maternal rurality and race/ethnicity. Findings demonstrate the disparities in virtual health care utilization, with pregnant individuals living in nonmetro rural areas reporting lower odds of using virtual mental health care (OR, 0.02; 95% CI, 0.10–0.81) and non-Hispanic other individuals showing lower odds of virtual mental health care use (OR, 0.18; 95% CI, 0.03–0.94) compared to their counterparts in large metro areas and non-Hispanic Whites. These results indicate a critical lack of accessibility and utilization of maternal mental health care, particularly in virtual settings. While the findings highlight existing disparities in access, COVID-19 underscored the critical need for flexible and accessible virtual mental health options, particularly for underserved communities. Addressing these disparities will require both immediate and sustained efforts to expand culturally sensitive, virtual, and community-based mental health resources that can endure beyond pandemic-specific needs. These disparities emphasize a broader issue within the health care system, where equitable access to maternal mental health services remains elusive to many, especially for those in rural areas and from diverse racial and ethnic backgrounds.

The insights gained from this study underscore the need for targeted maternal mental health care interventions that not only address the geographical and racial/ethnic disparities but also embrace the potential of virtual health care to overcome barriers to access. Policy reforms should focus on enhancing the availability and acceptability of virtual mental health services, ensuring that these interventions are inclusive and culturally sensitive. Moreover, our analysis calls for the development of specific, evidence-based strategies aimed at improving maternal mental health care for diverse populations. This includes increasing funding for mental health services in underrepresented communities, implementing community outreach programs to raise awareness about mental health issues and services among racial and ethnic minority groups, and integrating mental health care into routine prenatal and postpartum care to ensure early identification and treatment of mental health issues.

In conclusion, this study’s findings illuminate the complex interplay between residential rurality, race/ethnicity, and maternal mental health care accessibility and utilization, highlighting the need for comprehensive and tailored approaches to bridge the gap in maternal mental health care services. By focusing on targeted interventions, policy reforms, and the promotion of inclusive and culturally sensitive treatments, we can move closer to achieving equitable access to maternal mental health care services for all pregnant individuals.

## Data Availability

Data used in this research can be found on the official website of the National Survey on Drug Use and Health (NSDUH).

## References

[1] Bathija P, Syeda A. Making maternal mental health a priority. American Hospital Association. April 7, 2022. https://www.aha.org/news/blog/2022-04-07-making-maternal-mental-health-priority

[2] Trost SL, Beauregard JL, Smoots AN, et al. Preventing pregnancy-related mental health deaths: insights from 14 US Maternal Mortality Review Committees, 2008–17. Health Aff. 2021;40(10):1551–9. 10.1377/hlthaff.2021.00615. 10.1377/hlthaff.2021.00615 PMC11135281 34606354

[3] Sidebottom A, Vacquier M, LaRusso E, Erickson D, Hardeman R. Perinatal depression screening practices in a large health system: identifying current state and assessing opportunities to provide more equitable care. Arch Womens Ment Health. 2020. 10.1007/s00737-020-01035-x. 10.1007/s00737-020-01035-x PMC7929950 32372299

[4] Iturralde E, Hsiao CA, Nkemere L, et al. Engagement in perinatal depression treatment: a qualitative study of barriers across and within racial/ethnic groups. BMC Pregnancy Childbirth. 2021;21(1). 10.1186/s12884-021-03969-1. 10.1186/s12884-021-03969-1 PMC8284181 34271852

[5] Masters GA, Asipenko E, Bergman AL, et al. Impact of the COVID-19 pandemic on mental health, access to care, and health disparities in the perinatal period. J Psychiatr Res. 2021;137:126–30. 10.1016/j.jpsychires.2021.02.056. 33677216 10.1016/j.jpsychires.2021.02.056 PMC8084993

[6] Statz M, Bristow M. Addressing perinatal mood and anxiety disorders (PMADs) in rural places: a knowledge infrastructure. Wellbeing Space Soc. 2023;4:100131. 10.1016/j.wss.2023.100131.

[7] Morales DA, Barksdale CL, Beckel-Mitchener AC. A call to action to address rural mental health disparities. J Clin Transl Sci. 2020;4(5):463–7. 10.1017/cts.2020.42. 33244437 10.1017/cts.2020.42 PMC7681156

[8] Nidey N, Tabb KM, Carter KD, et al. Rurality and risk of perinatal depression among women in the United States. J Rural Health. 2019;36(1):9–16. 10.1111/jrh.12401. 31602705 10.1111/jrh.12401

[9] Yearby R, Clark B, Figueroa JF. Structural racism in historical and modern US health care policy. Health Aff. 2022;41(2):187–94. 10.1377/hlthaff.2021.01466. 10.1377/hlthaff.2021.01466 35130059

[10] Hansotte E, Payne SI, Babich SM. Positive postpartum depression screening practices and subsequent mental health treatment for low-income women in Western countries: a systematic literature review. Public Health Rev. 2017;38(1). 10.1186/s40985-017-0050-y. 10.1186/s40985-017-0050-y PMC5809911 29450075

[11] Salameh TN, Hall LA, Crawford TN, Staten RR, Hall MT. Racial/ethnic differences in mental health treatment among a national sample of pregnant women with mental health and/or substance use disorders in the United States. J Psychosom Res. 2019;121:74–80. 10.1016/j.jpsychores.2019.03.015. 30928211 10.1016/j.jpsychores.2019.03.015

[12] Mukherjee S, Trepka MJ, Pierre-Victor D, Bahelah R, Avent T. Racial/ethnic disparities in antenatal depression in the United States: a systematic review. Matern Child Health J. 2016;20(9):1780–97. 10.1007/s10995-016-1989-x. 27016352 10.1007/s10995-016-1989-x

[13] Meekins C. Supporting black women’s maternal mental health journey. IFDHE: American Hospital Association Institute for Diversity and Health Equity. July 19, 2022. https://ifdhe.aha.org/news/news/2022-07-19-supporting-black-womens-maternal-mental-health-journey#:~:text=Recognizing%20these%20issues%20increases%20the,Black%20women%20than%20White%20women

[14] Ceballos M, Wallace G, Goodwin G. Postpartum depression among African-American and Latina mothers living in small cities, towns, and rural communities. J Racial Ethn Health Disparities. 2016;4(5):916–27. 10.1007/s40615-016-0295-z. 10.1007/s40615-016-0295-z 27761728

[15] Kotlar B, Gerson E, Petrillo S, Langer A, Tiemeier H. The impact of the COVID-19 pandemic on maternal and perinatal health: a scoping review. Reprod Health. 2021;18(1):10. 10.1186/s12978-021-01070-6. 33461593 10.1186/s12978-021-01070-6 PMC7812564

[16] Liu J, Hung P, Alberg AJ, et al. Mental health among pregnant women with COVID-19-related stressors and worries in the United States. *Birth (Berkeley, Calif)*. Published online May 19, 2021. 10.1111/birt.12554. 10.1111/birt.12554 PMC8239832 34008216

[17] Duncan A, Herrera CN, Okobi M, Nandi S, Oblath R. Locked down or locked out? Trends in psychiatric emergency services utilization during the COVID-19 pandemic. J Health Serv Res Policy. Published online December 6, 2022:135581962211351. 10.1177/13558196221135119. 10.1177/13558196221135119 PMC9732494 36475326

[18] Hermann A, Fitelson EM, Bergink V. Meeting maternal mental health needs during the COVID-19 pandemic. *JAMA Psychiatry*. Published online July 15, 2020. 10.1001/jamapsychiatry.2020.1947. 10.1001/jamapsychiatry.2020.1947 32667662

[19] Ramsetty A, Adams C. Impact of the digital divide in the age of COVID-19. Journal of the American Medical Informatics Association. 2020;27(7). 10.1093/jamia/ocaa078. 10.1093/jamia/ocaa078 PMC7197532 32343813

[20] Saeed SA, Masters RM. Disparities in health care and the digital divide. Current Psychiatry Reports. 2021;23(9). 10.1007/s11920-021-01274-4. 10.1007/s11920-021-01274-4 PMC8300069 34297202

[21] Center for Behavioral Health Statistics and Quality. National survey on drug use and health population data. The Substance Abuse and Mental Health Services Administration. 2021. https://www.datafiles.samhsa.gov/dataset/national-survey-drug-use-and-health-2020-nsduh-2020-ds0001

[22] Center for Behavioral Health Statistics and Quality. National survey on drug use and health population data. The Substance Abuse and Mental Health Services Administration. 2022. https://www.datafiles.samhsa.gov/dataset/national-survey-drug-use-and-health-2021-nsduh-2021-ds0001

[23] USDA ERS - Rural-Urban Continuum Codes. www.ers.usda.gov. https://www.ers.usda.gov/data-products/rural-urban-continuum-codes/

[24] Rural-Urban Continuum Code - SEER datasets. SEER. https://seer.cancer.gov/seerstat/variables/countyattribs/ruralurban.html

[25] NIMHD minority health and health disparities research framework. National Institute on Minority Health and Health Disparities. 2018. https://nimhd.nih.gov/researchFramework 10.2105/AJPH.2018.304883 PMC6356129 30699025

[26] Jankovic J, Parsons J, Jovanović N, et al. Differences in access and utilization of mental health services in the perinatal period for women from ethnic minorities—a population-based study. BMC Med. 2020;18(1). 10.1186/s12916-020-01711-w. 10.1186/s12916-020-01711-w PMC7488566 32912196

[27] Rokicki S, Patel M, Suplee PD, D’Oria R. Racial and ethnic disparities in access to community-based perinatal mental health programs: results from a cross-sectional survey. BMC Public Health. 2024;24(1). 10.1186/s12889-024-18517-7. 10.1186/s12889-024-18517-7 PMC11031973 38643069

[28] Nana-Sinkam P, Kraschnewski J, Sacco R, et al. Health disparities and equity in the era of COVID-19. J Clin Transl Sci. 2021;5(1):e99. 10.1017/cts.2021.23. Published 2021 Mar 16. 34192054 10.1017/cts.2021.23 PMC8167251

[29] Ahad AA, Sanchez-Gonzalez M, Junquera P. Understanding and addressing mental health stigma across cultures for improving psychiatric care: a narrative review. Cureus. 2023;15(5):e39549. 10.7759/cureus.39549. Published 2023 May 26. 37250612 10.7759/cureus.39549 PMC10220277

[30] Taylor JK. structural racism and maternal health among Black women. J Law Med Ethics. 2020;48(3):506–17. 10.1177/1073110520958875. 33021163 10.1177/1073110520958875

[31] Fryer K, Lewis G, Munoz C, Stuebe AM. Identifying barriers and facilitators to prenatal care for Spanish-speaking women. N C Med J. 2021;82(1):7–13. 10.18043/ncm.82.1.7. 33397748 10.18043/ncm.82.1.7 PMC7927271

[32] Johnson MB. Prenatal care for American Indian Women. MCN Am J Matern Child Nurs. 2020. 10.1097/nmc.0000000000000633. Publish Ahead of Print. 32282338 10.1097/NMC.0000000000000633

[33] Millner UC, Maru M, Ismail A, Chakrabarti U. Decolonizing mental health practice: reconstructing an Asian-centric framework through a social justice lens. Asian Am J Psychol. 2021;12(4):333–45. 10.1037/aap0000268.

[34] Narayan S, Mok H, Ho K, Kealy D. “I don’t think they’re as culturally sensitive”: a mixed-method study exploring e-mental health use among culturally diverse populations. J Ment Health. 2023;32(1):241–7. 10.1080/09638237.2022.2091762. 35770901 10.1080/09638237.2022.2091762

[35] Declercq E, Feinberg E, Belanoff C. Racial inequities in the course of treating perinatal mental health challenges: results from listening to mothers in California. Birth. 2022;49(1):132–40. 10.1111/birt.12584. 34459012 10.1111/birt.12584 PMC9292331

[36] Haynes TF, Cheney AM, Sullivan JG, et al. Addressing mental health needs: perspectives of African Americans living in the rural south. Psychiatr Serv. 2017;68(6):573–8. 10.1176/appi.ps.201600208. 28142389 10.1176/appi.ps.201600208 PMC5646233

[37] Conteh N, Gagliardi J, McGahee S, Molina R, Clark CT, Clare CA. Medical mistrust in perinatal mental health. Harv Rev Psychiatry. 2022;30(4):238–47. 10.1097/HRP.0000000000000345. 35849741 10.1097/HRP.0000000000000345

[38] Morain SR, Fowler LR, Boyd JW. A pregnant pause: system-level barriers to perinatal mental health care. Health Promotion Practice. Published online June 21. 2022:152483992211013. 10.1177/15248399221101373 Published online June 21. 10.1177/15248399221101373 35726491

